# Cyberknife Radiosurgery for Prostate Cancer after Abdominoperineal Resection (CYRANO): The Combined Computer Tomography and Electromagnetic Navigation Guided Transperineal Fiducial Markers Implantation Technique

**DOI:** 10.3390/curroncol30090576

**Published:** 2023-08-28

**Authors:** Andrea Vavassori, Giovanni Mauri, Giovanni Carlo Mazzola, Federico Mastroleo, Guido Bonomo, Stefano Durante, Dario Zerini, Giulia Marvaso, Giulia Corrao, Elettra Dorotea Ferrari, Elena Rondi, Sabrina Vigorito, Federica Cattani, Franco Orsi, Barbara Alicja Jereczek-Fossa

**Affiliations:** 1 Division of Radiation Oncology, IEO European Institute of Oncology IRCCS, 20141 Milan, Italy; andrea.vavassori@ieo.it (A.V.); giovannicarlo.mazzola@ieo.it (G.C.M.); stefano.durante@ieo.it (S.D.); dario.zerini@ieo.it (D.Z.); giulia.marvaso@ieo.it (G.M.); giulia.corrao@ieo.it (G.C.); elettradorotea.ferrari@ieo.it (E.D.F.); barbara.jereczek@ieo.it (B.A.J.-F.); 2 Division of Interventional Radiology, IEO European Institute of Oncology IRCCS, 20141 Milan, Italy; giovanni.mauri@ieo.it (G.M.); guido.bonomo@ieo.it (G.B.); franco.orsi@ieo.it (F.O.); 3 Department of Oncology and Hemato-Oncology, University of Milan, 20141 Milan, Italy; 4 Department of Translational Medicine, University of Piemonte Orientale (UPO), 20188 Novara, Italy; 5 Unit of Medical Physics, IEO European Institute of Oncology IRCCS, 20141 Milan, Italy; elena.rondi@ieo.it (E.R.); sabrina.vigorito@ieo.it (S.V.); federica.cattani@ieo.it (F.C.)

**Keywords:** cyberknife, radiosurgery, prostate cancer, rectal cancer, abdominoperineal resection, Lynch Syndrome

## Abstract

In this technical development report, we present the strategic placement of fiducial markers within the prostate under the guidance of computed tomography (CT) and electromagnetic navigation (EMN) for the delivery of ultra-hypofractionated cyberknife (CK) therapy in a patient with localized prostate cancer (PCa) who had previously undergone chemo-radiotherapy for rectal cancer and subsequent abdominoperineal resection due to local recurrence. The patient was positioned in a prone position with a pillow under the pelvis to facilitate access, and an electromagnetic fiducial marker was placed on the patient’s skin to establish a stable position. CT scans were performed to plan the procedure, mark virtual points, and simulate the needle trajectory using the navigation system. Local anesthesia was administered, and a 21G needle was used to place the fiducial markers according to the navigation system information. A confirmatory CT scan was obtained to ensure proper positioning. The implantation procedure was safe, without any acute side effects such as pain, hematuria, dysuria, or hematospermia. Our report highlights the ability to use EMN systems to virtually navigate within a pre-acquired imaging dataset in the interventional room, allowing for non-conventional approaches and potentially revolutionizing fiducial marker positioning, offering new perspectives for PCa treatment in selected cases.

## 1. Introduction

Prostate cancer (PCa) ranks as the second most-prevalent malignancy among males worldwide [1], with an estimated number of 268,490 new prostate cancer cases in 2022, accounting for the 27% of all new cancer diagnoses according to the latest American Cancer Society report [2].

In therapeutical workup, radiation therapy (RT) has progressively emerged as a well-established treatment modality in localized PCa [3], with the use of dose-escalated intensity-modulated radiation therapy (IMRT). To ensure a precise irradiation of the target volume, different treatment techniques and different image-guidance systems have now become available, leading to the use of image-guided RT (IGRT) as a gold-standard. Further recent advances in imaging, treatment-planning and delivery systems have increased interest in hypofractionation, leading to the use of fewer large doses per fraction delivered in a very short treatment time [4,5,6]. Similar treatment approaches in the case of RT with more than 4 Gy per fraction have been defined as ultra-hypofractionated regimens [7], showing biochemical control comparable with the one obtained by conventional fractionation with similar toxicity profiles [8].

The Cyberknife (CK) system (Accuray, Inc., Sunnyvale, California) has been widely adopted for the delivery of PCa ultra-hypofractionated treatments [9] thanks to its peculiar combination of an arm-based robotic system carrying a compact X-band linear accelerator, while integrating radiographic imaging (orthogonal KV X-ray system) and motion feedback trackers. This system has shown comparable treatment efficacy to standard linear accellerators, with reductionin side effects [10], but further studies are needed to better address the differences in dose distribution to both target and normal tissues [11].

For the accurate tracking of target volume movements during treatment, CK could rely on from 3 to 6 radio-opaque gold fiducial markers, usually placed within the prostate by rectal ultrasound-guidance. The fiducials play the role of a surrogate position for the prostate within the reference frame of the planned CT scan and, following Accuray’s guidelines, their role is to map translations and rotations following six degrees of freedom [12]. Common issues related to improper fiducials’ placement include a distance of less than 2 cm between each fiducial and the absence of colinear placement within the orthogonal imaging plane. Holmes et al. proposed a modified implantation protocol for fiducial markers, which involved four fiducial markers implanted in the postero-lateral aspect of the prostate, following a single coronal plane. This approach has shown a significant increase in the ability to accurately track prostate motion during CK treatment [13].

Electromagnetic navigation (EMN) has emerged as a transformative technology within the realm of medical imaging, particularly in the context of CT-guided biopsies [14]. By utilizing specialized electromagnetic sensors and markers, physicians are able to track the real-time position and orientation of biopsy needles or instruments within the patient’s body. This real-time feedback empowers medical professionals to navigate complex anatomical structures with greater confidence, thereby reducing the risk of inadvertent damage to surrounding tissues. Furthermore, the consolidated use of EMN with CT imaging provides a synergistic effect that combines the detailed anatomical information provided by CT scans with the dynamic guidance of EMN, resulting in an advanced and highly targeted approach to performing biopsies [15,16]. As EMN technology continues to evolve and its integration with CT becomes more seamless, the potential for its wider adoption in various diagnostic and interventional procedures, such as fiducial marker implantation, appears promising.

We present a complex case study of a Lynch syndrome patient with localized PCa who underwent ultra-hypofractionated CK therapy following prior surgery and chemo-radiotherapy treatment for rectal cancer and subsequent abdominoperineal resection due to local recurrence.

Given the unique anatomical circumstances, fiducial markers were strategically positioned within the prostate under the guidance of computed tomography (CT) and electromagnetic navigation. The placement of fiducial markers using the present technique could represent a new perspective for PCa treatment in selected cases.

## 2. Case Report

Here, we report the case of a 60-year-old Caucasian man with LS, admitted to our Radiotherapy Department for the treatment of a localized intermediate–high prostate cancer in January 2023.

LS is an autosomal dominant genetic disorder characterized by an increased risk of developing colorectactal cancer (CRC) and, secondly, endometrial, ovarian, gastric and urinary tract cancers, among others [17,18,19]. LS is considered the most common heritable cancer syndrome, initially referred to as hereditary nonpolyposis colorectal cancer (CRC), which affects 1 in 400 people and accounts for the 3% of the total number of new CRC diagnosis [20]. This is due to germ-line mutations in MLH1, MSH2, MSH6, and PMS2 mismatch-repair genes, which lead to microsatellite instability in the accumulation of errors or variations [21].

In 1996, the patient underwent a radical transabdominal resection with colo-anal anastomosis and received adjuvant chemo-radiotherapy up to a total dose of 45.9 Gy (1.7 Gy per fraction; 27 fractions) for a rectal adenocarcinoma staged as pT2 N0 M0. In 2005, he subsequently underwent an abdominal perineal resection (APR) and permanent colostomy due to the recurrence of colorectal cancer classified as rpT2 N0 M0.

Additionally, in 2017, the patient underwent a right nephrectomy for a high-grade transitional cell carcinoma and, in 2018, a Transurethral Resection of Bladder Tumor (TURBT), followed by the instillation of Mitomycin C for a non-invasive, low-grade papillary urothelial carcinoma.

In 2021, the patient underwent a total colectomy due to the diagnosis of a right colon adenocarcinoma classified as pT4bN0 G3.

In November 2022, due to an increasing Prostate-Specific Antigen (PSA) level of up to 17.2 ng/mL, he performed CT-guided prostate biopsy, leading to the diagnosis of an adenocarcinoma with a Gleason Score 3 + 4 = 7 (Grade 2 ISUP 2015), and T2b N0 M0 stage was confirmed.

The patient’s case was presented at the multidisciplinary tumour board and, considering the risks associated with the previous surgeries, total prostatectomy was deemed contraindicated. Instead, reirradiation using a radiosurgical approach with CK combined with androgenic deprivation therapy was proposed to minimize the risk of urinary and gastrointestinal toxicity, along with the potential risk of secondary tumorigenesis associated with LS [22].

In March 2023, in collaboration with the interventional radiology unit, four dedicated gold fiducial markers were implanted in the prostate using a transperineal approach, using two pre-loaded needles guided by CT and a dedicated electromagnetic navigation guided (CT-NavigationTM, Imactis, Saint-Martin-d’Hères, France). The latter is an independent setup with a dedicated monitor, an integrated software, an electromagnetic fiducial marker/field generator, and a sensor that is applied to the device whose direction is being estimated.

**Figure 1:**
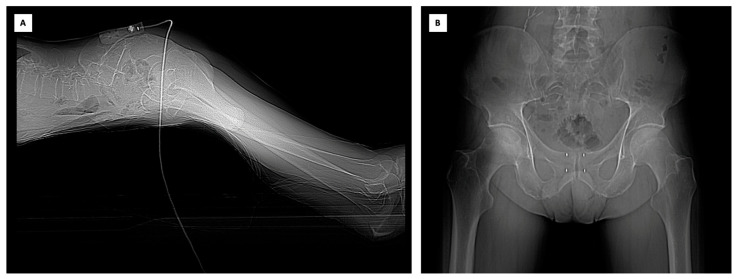
(**A**) The electromagnetic fiducial marker/field generator applied to the skin of the patient in prone position. (**B**) The CT confirmed the correct positioning of the markers.

To establish a stable position that is as close as possible to the procedure area, an electromagnetic fiducial marker, which also acts as electromagnetic-field-generator, was placed on the patient’s skin. The patient was positioned prone with a pillow under the pelvis to facilitate transperineal access. A CT scan was performed, encompassing the planned entry point, the prostate, and the electromagnetic fiducial marker. Virtual points were marked on the acquired images, and the sensor was used to navigate the preacquired CT volume, simulating the needle’s trajectory from the skin to the desired final marker position. It was fundamental to ensure that the four markers were positioned as parallel as possible for the subsequent stereotactic radiation therapy. Subsequently, using a 21G needle, local anaesthesia was administered in the periprostatic tissue. The needle was placed according to the virtual navigation system information and a new CT scan was acquired to confirm the correct positioning. Following the same method, the fiducial markers were ultimately placed in the prostate, and a final CT scan confirmed their correct positioning (Figure 1).

The implantation procedure of radio-opaque gold markers was safe, with no acute side effects such as perineal pain, hematuria, dysuria or hematospermia.

Three weeks after the implantation of the fiducials, a simulation CT scan of the pelvis and a prostatic MRI were performed. The clinical target volume of the prostate (CTV-P), which includes the entire prostate and the proximal one-third of seminal vesicles, as well as the organs at-risk (bladder, penis, penile bulb, testis, femoral heads, cauda, rectum, anal canal and abdominal sac) were delineated using the dedicated treatment-planning system (TPS).

**Figure 2:**
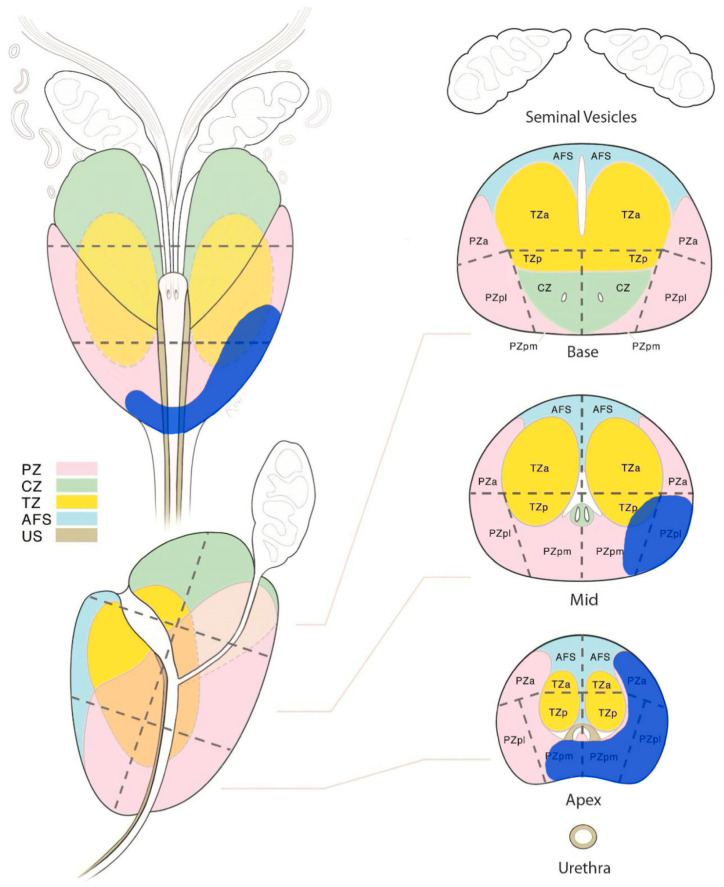
The multi-parametric magnetic resonance imaging diagram, showing the dominant intraprostatic lesion (blu area) in the lower and medium/middle third of the prostate. PZ: peripheral zone, CZ: central zone, TZ: transizional zone, AFS: anterior fibromuscolar stroma, US: urethral sphincter, a: anterior, p: posterior, m: medial, l: lateral.

The delineation of the CTV of the dominant intraprostatic lesion (CTV-DIL) was performed by the integration of multiparametric magnetic resonance imaging (mpMRI) sequences with the planned CT simulation in the Treatment Planning System (TPS). The mpMRI sequences, encompassing various imaging contrasts such as T2-weighted, diffusion-weighted, and dynamic contrast-enhanced sequences, provide detailed information about the tumor’s morphology, vascularity, and cellular density. These sequences offer a multi-dimensional perspective, allowing for the identification of the DIL with high accuracy (Figure 2).

The planning target volume of the prostate (PTV-P) was established by applying a systematic expansion of 5 mm in all the directions, except for a 3 mm expansion posteriorly. Similarly, for the planning target volume of the dominant intraprostatic lesion (PTV-DIL), a uniform expansion of 3 mm in all directions was added to CTV-DIL [23].

**Figure 3:**
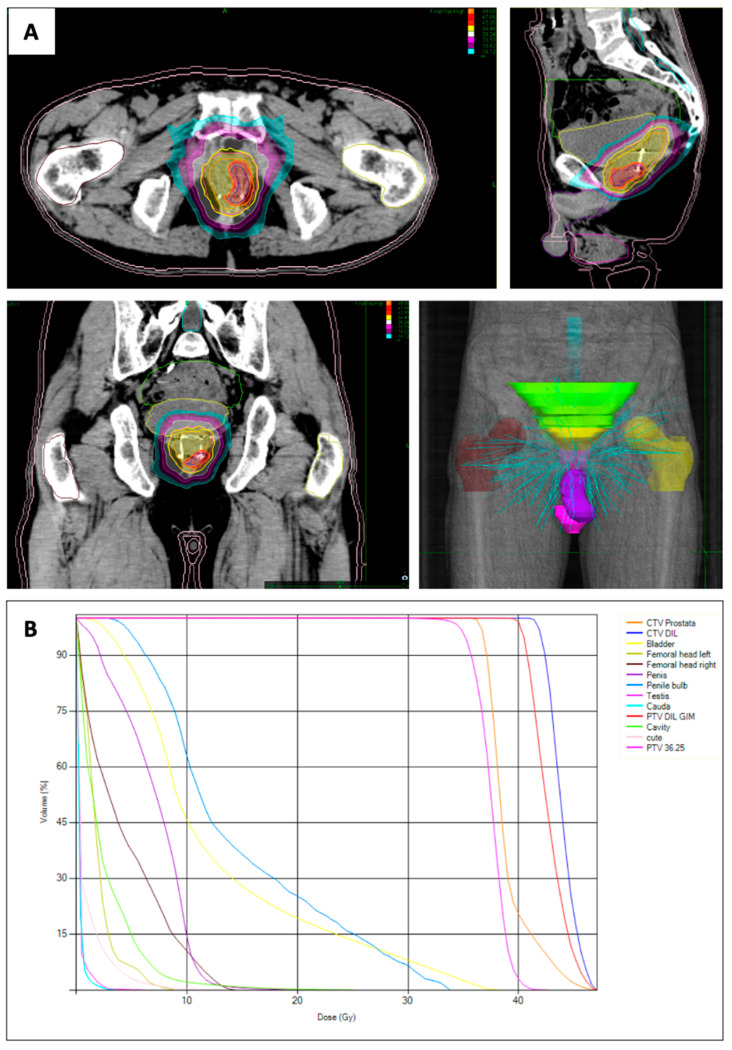
(**A**) Dose distribution with the dominant intraprostatic lesion (DIL) in the left peripheral-apex side of the prostate. (**B**) Dose–volume histograms of targets (planning target volume (PTV) of the dominant intraprostatic lesion and PTV of the prostate) and organ at risk. Volume of the clinical target volume (CTV) of the prostate: 48.7cc, Volume CTV DIL: 4.3cc. Dose Max 47.06 Gy.

In accordance with the treatment strategy, the PTV-P was subjected to a total radiation fose of 36.25 Gy, which was delivered through a fractionation schedule of 7.25 Gy per fraction. The PTV-DIL received an escalated radiation dose of 40 Gy, administered in 8 Gy fractions (Table 1). The treatment course spanned 11 days, with a fraction every other day. The prescribed dose was normalized to the 85% isodose to ensure consistent and accurate dosimetry across both planning target volumes (Figure 3).

According to the linear quadratic model, the equivalent biological dose (EQD) given at 2 Gy per fraction (EQD2 = D[α/β + d]/[α/β + 2], where D is the total dose given at the dose per fraction d) for PTV-P and PTV-DIL was 90.6 Gy and 108.6, respectively, assuming the α/β value of 1.5 Gy.

After a follow-up of one month, the patient had no severe acute urinary toxicity, such as bladder outlet obstruction, gross hematuria, or urgency, as per the Common Terminology Criteria for Adverse Events criteria.

The research participant granted informed consent for research purposes, ensuring compliance with ethical standards. Moreover, the present manuscript meticulously adheres to the CARE guidelines, upholding the principles of transparency and rigor in reporting.

## 3. Discussion

Over the past few decades, the literature has demonstrated a growing interest in reirradiation cases [24,25]. Due to factors such as accumulated prior dose and anatomical changes, these cases require more complex planning. In the presented case, the patient’s distinctive anatomical circumstances, arising from prior surgeries and radiation treatment, necessitated a tailored therapeutic strategy. Consequently, the utilization of ultra-hypofractionated CK therapy alongside androgen deprivation therapy was deemed to be the optimal approach. After a thorough assessment of the patient’s medical history and considering the time that elapsed since the previous rectal cancer treatment, the treatment team ascertained that the current dosage and approach hold promising curative potential for management of the localized PCa.

The CK system has demonstrated a wide range of application for treating primary or recurrent tumors, as well as small metastatic lesions, providing excellent local control and minimal toxicity, even in cases of previous surgery or radiotherapy [26]. The system utilizes integrated digital X-ray imaging, enabling the use of bone landmarks or gold fiducial markers, which are used to set up the patient and track the position of the target volume throughout the entire treatment course.

Radio-opaque markers could be inserted using different approaches. In general, image-guided procedures such as percutaneous biopsies, ablations or marker placements rely heavily on the accuracy of imaging visualization. Ultrasound (US) is commonly used to guide interventions in the abdomen, mainly due to its real-time capabilities and its ability to select non-axial approaches. However, finding an adequate acoustic window might not always be easy. CT provides a broader field of view and is not limited by the presence of air or bones, but requires an axial or near-axial approach and lacks real-time capabilities. Technological advancements now allow for the real-time fusion of US and CT images, enhancing the guidance of percutaneous procedures and overcoming the limitations of each individual imaging modality [27,28].

In addition, the application of an EMN facilitates virtual navigation within a pre-acquired imaging dataset right within the interventional room, thereby enabling unconventional approaches. Previous research has demonstrated that employing such navigation systems for CT-guided interventions yields remarkable needle-placement accuracy, even in case of out-of-plane trajectories [29]. This augmented precision allows for the feasibility of oblique trajectories, thereby expanding the spectrum of possible needle pathways beyond what conventional US or CT guidance would offer [30]. Crucially, the incorporation of oblique trajectories into the procedural repertoire broadens the horizons of feasible pathways, which are otherwise limited by conventional guidance techniques. As a result, the navigation system not only enhances the accuracy of the procedural maneuvers but also instills greater confidence in the operator’s actions. This dual effect is anticipated to concomitantly decrease radiation exposure, intervention duration, and the severity of the intervention’s impact [31].

There have only been a few reports on the use of CK after APR. In 2010, Thariat et al. published a case report about the use of CK stereotactic radiotherapy for a woman with bladder cancer that developed after previous chemoradiotherapy and abdominoperineal resection for a rectal cancer. The patient received a total dose of 24 Gy in six fractions to the TURB area, with gold markers placed under urethrocystoscopy guidance. Two years following the conclusion of the treatment, the patient’s overall conditions remained stable, with an absence of hematuria, diarrhoea, or pain, and no evidence of disease, confirmed by cystoscopic examination and abdominopelvic CT [32].

In 2016, Dagoglu et al. conducted a retrospective study involving a cohort of seven rectal cancer patients who underwent treatment with CK following induction chemoradiation and surgery due to positive or close margins. In this particular series, fiducial seeds were placed at surgery, and the median stereotactic dose was 25 Gy in five fractions. The results of this study demonstrated both the safety and efficacy of this approach within the scope of a small retrospective series. However, to establish a more comprehensive understanding of its potential benefits and limitations, a larger-scale evaluation would contribute to a more thorough comprehension of its implementation [33].

Within the context of the current literature, our case report sheds light on a novel approach—the utilization of CK as a solitary therapeutic avenue for irradiating the prostate in cases of prostate cancer, subsequent to pelvic irradiation and APR. However, the present study represents an initial step in exploring this innovative technique’s feasibility and potential. To draw robust conclusions and validate its clinical efficacy, it is imperative that further investigations be conducted with a larger and more diverse patient cohort.

With an increased number of patients, it is likely that a clearer understanding of the technique’s benefits, limitations, and long-term outcomes can be attained. In addition, a broader patient pool would enable a more accurate assessment of its safety profile and applicability across a spectrum of clinical scenarios.

## Figures and Tables

**Table 1:** Prescription doses (D) table of targets: planning target volume of the dominant intraprostatic lesion (PTV-DIL) and planning target volume of the prostate (PTV-P).

Dose Target	Goal	Results
PTV-DIL (40 Gy)		
D0.03_cc_	<116%	117.00%
D98%		100.50%
D95%	>95%	101.30%
D2%	<114%	103.40%
D50% (Median)		106.30%
**PTV-P (36.25 Gy)**		
D 0.03_cc_	<114%	115.30%
D98%		95.20%
D95%	>95%	97.10%
D2%	<104%	110.9%
D50% (Median)		103.70%

## Data Availability

The data presented in this study are available on request from the corresponding author. The data are not publicly available due to protection of patient privacy.
